# A290 PHENOTYPE AND OUTCOME OF PATIENTS HOSPITALIZED FOR ACUTE PANCREATITIS IN A TERTIARY PEDIATRIC CENTER

**DOI:** 10.1093/jcag/gwac036.290

**Published:** 2023-03-07

**Authors:** T Li, S Blain, C Korman, A David, M Mohamed, D Elhaoua, F Alvarez, C Deslandres, M Dirks, U Halac, K Grzywacz, M Lallier, P Jantchou

**Affiliations:** 1 Gastroentérologie, Centre hospitalier universitaire Sainte-Justine, Montreal; 2 Université d'Ottawa, Ottawa, Canada

## Abstract

**Background:**

A recent meta-analysis of 48 studies, showed an equal prevalence of AP (16%) among the following etiologies; systemic disease, alcohol, medication, genetics, gallstones and infection in North American hospitalized and ambulatory pediatric patients. However, data on the epidemiology of severe pediatric acute pancreatitis (AP) in Canada are lacking.

**Purpose:**

We aim to evaluate the clinical presentation, etiologies, comorbidities and outcome of pediatric patients with AP admitted to a tertiary hospital in Quebec, Canada.

**Method:**

A retrospective observational cohort study (January 2014-December 2021) was performed at the CHU Sainte-Justine. Descriptive analyses were performed with SAS statistical softwar

**Result(s):**

Among the 214 patients included (110 (51%) males), 58 (27.1%) were already hospitalized at time of AP diagnosis (AP as secondary diagnosis) while 156 (72.9%) were admitted from the emergency room mainly with a presentation of abdominal pain (AP as primary diagnosis). Thirty-two patients (15.0%) were transferred to the ICU due to hemodynamic instability or respiratory failure. Comorbidities included cancer (38 patients (17.7%)), obesity (17 (7.9%)) and inflammatory bowel disease (15 (7.0%)). The three most commonly identified etiologies were medication (19.6%), biliary disease (16.3%) and infection (14,9%). Despite extensive investigations, 26.2% of cases were idiopathic. The main complications were, ascites (48 patients (22.4%)), necrotic pancreatitis (10 (4.6%)) and pancreatic pseudocyst (10 (4.6%)). The median duration of hospitalization for AP as a primary diagnosis was 4 days (interquartile range (IQR) 2-7) as compared to 22 (11-37) for AP as a secondary diagnosis.

**Conclusion(s):**

Approximately one third of hospitalized patients had an underlying condition requiring treatments that could cause AP, which explains the high prevalence of drug-induced AP in this report. The longest hospitalizations were associated with AP as secondary diagnosis. Ongoing work will identify factors associated with disease severity and outcome in particular in primary AP.

**Please acknowledge all funding agencies by checking the applicable boxes below:**

None

**Disclosure of Interest:**

None Declared

